# Carbon-ion radiotherapy for hepatocellular carcinoma with major vascular invasion: a retrospective cohort study

**DOI:** 10.1186/s12885-024-12154-4

**Published:** 2024-03-26

**Authors:** Takashi Kaneko, Hirokazu Makishima, Masaru Wakatsuki, Yuichi Hiroshima, Toshiaki Matsui, Shigeo Yasuda, Naomi Nagatake Okada, Kenji Nemoto, Hiroshi Tsuji, Shigeru Yamada, Masaru Miyazaki

**Affiliations:** 1 QST Hospital, National Institutes for Quantum Science and Technology, 4-9-1 Anagawa, Inage-Ku, Chiba, 263-8555 Japan; 2 Department of Radiology, Division of Radiation Oncology, Yamagata University, Faculty of Medicine, Yamagata, Japan; 3 Department of Radiation Oncology, University of Tsukuba, Tsukuba, Japan; 4 Department of Radiation Oncology, Ibaraki Prefectural Central Hospital, Ibaraki Cancer Center, Kasama, Japan; 5 Department of Radiation Oncology, Saitama Cancer Center, Saitama, Japan; 6 Department of Radiation Oncology, Chiba Rosai Hospital, Chiba, Japan; 7 Mita Hospital, International University of Health and Welfare, Tokyo, Japan

**Keywords:** Liver cancer, Particle therapy, Major vascular invasion, Local control

## Abstract

**Background:**

Macroscopic vascular invasion (MVI) significantly impacts survival in patients with hepatocellular carcinoma (HCC), warranting systemic therapy over locoregional therapy. Despite novel approaches, HCC with MVI has a poor prognosis compared to early-to intermediate-stage HCC. This study aimed to evaluate the safety and efficacy of carbon-ion radiotherapy (C-ion RT) for HCC characterized by MVI.

**Methods:**

This retrospective cohort study evaluated HCC patients with MVI treated using C-ion RT with a dose of 45.0–48.0 Gy/2 fractions or 52.8–60.0 Gy/4 fractions between 1995 and 2020 at our institution in Japan. We analyzed the prognostic factors and rates of local recurrence, survival, and adverse events. The local recurrence rate was determined using the cumulative incidence function, with death as a competing event. Survival rates were determined using the Kaplan–Meier method. The log-rank test for univariate analysis and the Cox proportional hazards model for multivariate analysis were used to compare subgroups.

**Results:**

In total, 76 patients with a median age of 71 years (range, 45–86 years) were evaluated. Among them, 68 had Child–Pugh grade A while eight had grade B disease. In 17 patients, the vascular tumor thrombus reached the inferior vena cava or main trunk of the portal vein. Over a median follow-up period of 27.9 months (range, 1.5–180.4 months), the 2-year overall survival, progression-free survival, and local recurrence rates were 70.0% (95% confidence interval [CI]: 57.7–79.4%), 32.7% (95% CI: 22.0–43.8%), and 8.9% (95% CI: 1.7–23.5%), respectively. A naïve tumor and a single lesion were significant prognostic factors for overall survival in the univariate analysis. Albumin-bilirubin grade 1 and a single lesion were independent prognostic factors in the multivariate analysis. Overall, four patients (5%) experienced grade 3 late adverse events, with no observed grade 4 or 5 acute or late adverse events.

**Conclusions:**

C-ion RT for HCC with MVI showed favorable local control and survival benefits with minimal toxicity.

**Supplementary Information:**

The online version contains supplementary material available at 10.1186/s12885-024-12154-4.

## Background

Primary liver cancer, mostly hepatocellular carcinoma (HCC), is the sixth most frequently diagnosed cancer and the fourth leading cause of death worldwide [1]. The American Association for the Study of the Liver Disease/Barcelona Clinic for Liver Cancer (AASLD/BCLC) staging system and treatment guidelines recommend various treatment modalities and combination therapies according to cancer stage and liver function [2]. Macroscopic vascular invasion (MVI) has a huge impact on the treatment outcomes and survival of patients with HCC, in addition to tumor size, number of tumors, and liver function [3–7]. The AASLD/BCLC staging system and treatment guidelines classify HCC with MVI as advanced-stage disease and recommends systemic therapy [2]. Although atezolizumab + bevacizumab combination immunotherapy has achieved better survival than sorafenib alone, the prognosis remains poor, with a median survival of only 20 months [8]. The reasons for this are a low complete response rate to systemic therapy and rapid disease progression.

Given that tumor thrombi are often the leading cause of death in these patients, local therapy could play an important role, especially with the improved overall survival (OS) benefits of new systemic treatment options. Surgical resection has been explored in patients with MVI, showing longer survival than nonsurgical treatment in cases with vascular invasion limited to the first-order branch of the portal vein or the major hepatic vein [9, 10]. However, its suitability depends on disease progression and general condition of the patient, and may not be indicated for all HCC with MVI.

Different treatment modalities, such as radiotherapy, are used in unresectable cases. Several studies have reported treatment outcomes of photon radiotherapy for unresectable HCC with MVI [11–13]. Although photon radiotherapy demonstrates a better prognostic benefit than sorafenib in patients with HCC and MVI, the OS remains unsatisfactory, with a median of 10.9 months (versus 4.8 months for sorafenib) [14]. Local recurrence (LR) poses a challenge due to the physical limitations of X-rays [14]. Meanwhile, particle therapy, including carbon-ion radiotherapy (C-ion RT), offers better dose distribution properties owing to the Bragg peak and reduced lateral scattering. This enables a higher prescribed dose for HCC compared to photon radiotherapy [15]. Previous studies have reported that the irradiation volume of the liver is lower with C-ion RT compared to that of stereotactic body radiotherapy (SBRT) or intensity-modulated radiotherapy [16, 17]. Several articles, including prospective studies, have reported promising clinical outcomes of C-ion RT for HCC, and its potential effectiveness in HCC with MVI [18–21].

This study aimed to evaluate the safety and efficacy of C-ion RT for the treatment of HCC with MVI.

## Methods

### Study design and ethics

This retrospective study was approved by the Certified Review Board of the National Institutes for Quantum Science and Technology (No. 20–046) and was conducted in accordance with the Declaration of Helsinki. All patients provided informed consent, authorizing the use of their clinical information for research purposes.

### Participants

We evaluated patients who underwent C-ion RT for HCC at our institution between June 1995 and March 2020. Patients were identified using the institutional database and were enrolled based on following inclusion criteria: (1) C-ion RT with 45.0–48.0 Gy/2 fractions or 52.8–60.0 Gy/4 fractions; (2) confirmation of vascular invasion to the first-order branch of the portal vein or/and major hepatic vein confirmed by dynamic contrast-enhanced computed tomography (CT) or magnetic resonance imaging (MRI); (3) for patients with multiple lesions, C-ion RT targeted the tumor thrombus and its feeding tumor, while all remaining lesions received various local therapies including but not limited to C-ion RT; (4) N0M0 status; (5) ineligibility for, or refusal of systemic therapy; (6) definitive treatment intent; (7) Eastern Cooperative Oncology Group performance status of 0 to 2; (8) controllable ascites; and (9) Child–Pugh grade A or B disease. Patients who previously underwent irradiation for the same lesion and those with active cancers other than HCC were excluded. Of the 750 patients identified 76 eligible patients were evaluated.

### Carbon-ion radiotherapy

The beam delivery and calculation models have been documented elsewhere [15, 22, 23]. Briefly until 2015, beam delivery employed passive scattering methods, while treatments in subsequent years used spot scanning. Microdosimetric kinetic models for passive scattering and modified microdosimetric kinetic models for spot scanning were used for the calculations. Initially, the beam angles were fixed at vertical and horizontal angles until 2017. However, since 2017, the rotating-gantry beam system has been operational, allowing irradiation from any angle within 360 degrees [24]. Based on report 93 from the International Commission on Radiation Units and Measurements, the relative biological effectiveness (RBE)-weighted doses of C-ion RT, defined as the absorbed dose multiplied by the RBE of carbon ions, are expressed in Gy. All prescribed doses of C-ion RT in this study are presented as RBE-weighted doses.

Before therapeutic planning, fiducial metallic markers were implanted near the tumor in all the patients to ensure precise treatment positioning. The irradiation fields were established using a three-dimensional therapy plan based on the CT images. Radiation treatments were planned on a CT-based three-dimensional treatment planning system using the HIPLAN software program (NIRS, Chiba, Japan) or XiO-N (ELEKTA, Stockholm, Sweden, and Mitsubishi Electric, Tokyo, Japan) [25]. The gross tumor volume (GTV) was defined using dynamic contrast-enhanced CT and MRI. The clinical target volume was defined as a margin of 5–10-mm from the GTV and an additional 10-mm margin alongside the vessel for the tumor thrombus. A 3–5-mm margin was added to compensate for internal motion, and another 2–3-mm margin was added for setup error to form the planning target volume (PTV). The future minimum remnant liver volume (volume receiving less than 30 Gy) after C-ion RT was set at 500 cm^3^ [26, 27].

To accurately reproduce the target position, a low-temperature thermoplastic sheet (Shellfitter, Kuraray, Osaka, Japan), customized cradle (Moldcare, Alcare, Tokyo, Japan), and respiratory-gated irradiation system were used for CT planning and radiotherapy [28]. The radiation field was confirmed and corrected using orthogonal fluoroscopy and radiography immediately before treatment. Both bone and metallic markers were allowed a tolerance of up to 3 mm. In cases with larger misalignments, radiation oncologists re-evaluated the treatment plan and determined whether it was sufficiently robust. The prescribed dose of C-ion RT for HCC initially began at 49.5 Gy/15 fractions in a phase I/II clinical trial. Following subsequent dose escalation and hypofractionation trials, our institution currently employs two protocols of 48 Gy/2 fractions and 60 Gy/4 fractions [18, 19, 29]. A previous report demonstrated that there was no difference in local control between the two-fraction and four-fraction protocols [30]. A four-fraction protocol was selected for cases that did not meet the criteria of previous clinical trials on two-fraction protocol including, proximity to the intestinal tract or the presence of liver dysfunctions [19, 29].

### Follow-up and evaluation of clinical outcomes

Following C-ion RT for HCC, patients were on follow-up with contrast-enhanced CT or MRI of the liver region every 3 months for the first 2 years and 3 to 6 months thereafter. Blood tests were performed one month after C-ion RT and at each subsequent imaging examination. LR was defined as the evidence of tumor regrowth in the PTV, including the PTV margin. The progression-free status was defined as the absence of LR, intrahepatic metastasis, or distant metastasis. Acute and late adverse events were classified according to the Common Terminology Criteria for Adverse Events, version 4.0.

### Statistical analysis

All survival periods were calculated from the first day of C-ion RT. The LR rate was determined using the cumulative incidence function with death as a competing event. The progression-free survival (PFS) and OS rates were determined using the Kaplan–Meier method. For univariate analyses, log-rank tests were used to compare OS among the subgroups. Continuous variables, such as age and maximum tumor diameter, were divided into two groups based on median values. For tumor markers, 400 ng/mL for alpha-fetoprotein and 100 mAU/mL for des-gamma carboxyprothrombin were set as cutoff values, in accordance with a previous report [6]. The entire cohort was divided into two categories based on the treatment start dates: the first and the second halves, with 2008 as the pivotal year when sorafenib was introduced [31]. The risk factors previously reported, including age, sex, Child–Pugh grade, albumin-bilirubin grade (ALBI grade), tumor status, number of lesions, and maximum tumor diameter, were included in the multivariate analysis using the Cox proportional hazards model [3–7]. Patient and tumor factors were compared between the two groups using a *t*-test for continuous variables and a *chi*-square test for categorical variables. All statistical analyses were performed using the R software (http://www.rproject.org/). All statistical tests were two-sided, and statistical significance was set at *p* < 0.05.

## Results

### Patient, tumor, and treatment characteristics

The median patient age was 71 years (range: 45–86 years). All tumors were classified as T4N0M0 according to the 8th edition of the TNM staging system (Union for International Cancer Control, 2017) and were in an advanced-stage according to the AASLD/BCLC staging system. Patient characteristics, tumor characteristics, and treatment details are summarized in Tables 1 and 2. Regarding tumor status, seven patients had regional recurrence in the liver, and 29 patients had LR after various previous treatments. Twelve patients had multiple lesions, of which eight were treated with multiple treatment modalities. The most commonly prescribed dose was 52.8 Gy/4 fractions (49%).

**Table 1:** Patient characteristics (*n* = 76)

Characteristic	n
Age (years),	
median (range)	71 (45–86)
Sex	
Male	58
Female	18
Performance status	
0	55
1	18
2	3
Etiology	
Hepatitis C virus	41
Hepatitis B virus	20
Alcoholic	4
Drug-induced	1
Non-B non-C	10
Child–Pugh score	
5	47
6	21
7	6
8	2
ALBI grade	
1	47
2a	10
2b	19
AFP (ng/mL),	
median (range)	79.6 (1.1–140,000.0)
DCP (mAU/mL),	
median (range)	126.5 (12.0–60725.0)

*Abbreviations*: *ALBI* *grade* Albumin-bilirubin grade, *AFP* Alpha-fetoprotein, *DCP* Des-gamma carboxyprothrombin

**Table 2:** Tumor and treatment characteristics (*n* = 76)

**Characteristic**	**n**
Tumor status	
Naïve	40
Reginal or local recurrence	36
Number of lesions	
1	64
2	5
3	4
4	3
Maximum tumor diameter (cm),	
median (range)	4.6 (1.5–13.0)
Portal vein invasion	
≤ 1	46
2	9
3	16
4	5
Hepatic vein invasion	
≤ 1	10
2	54
3	12
Bile duct invasion	
≤ 1	72
2	0
3	4
4	0
Protocol dose (BED_10_)	
45.0 Gy/2 fractions (146.3 Gy)	10
48.0 Gy/2 fractions (163.2 Gy)	22
52.8 Gy/4 fractions (122.5 Gy)	37
60.0 Gy/4 fractions (150.0 Gy)	7

*Abbreviations*: *BED* Biologically effective dose

### Efficacy

**Fig. 1:**
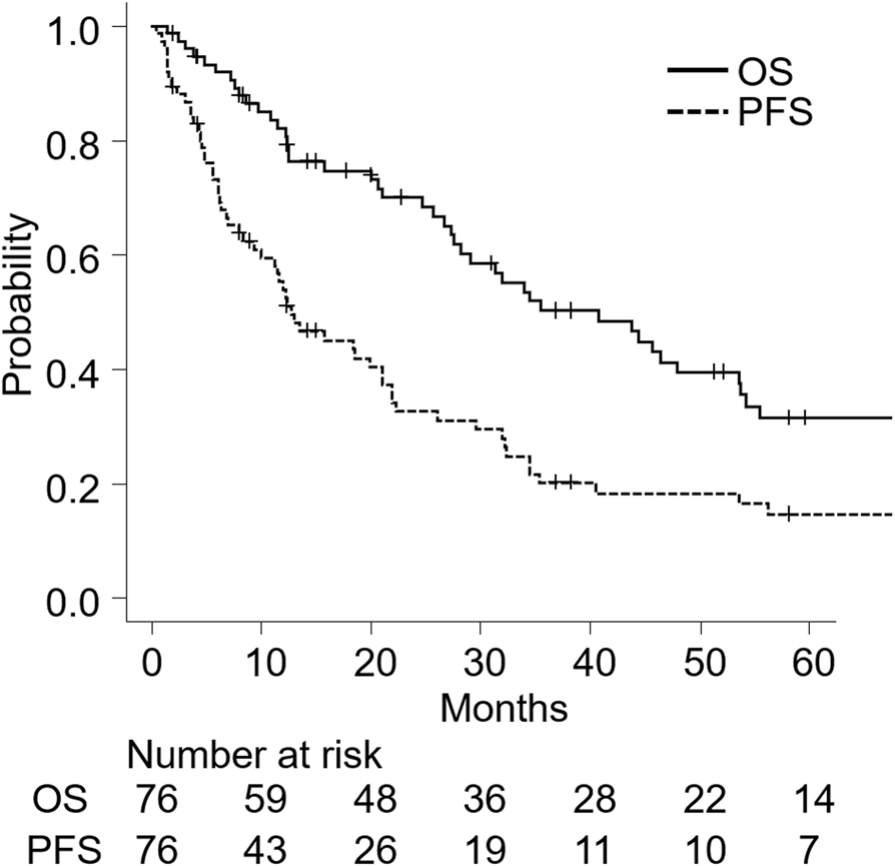
OS and PFS curves after carbon-ion radiotherapy. Legend: OS (full line) and PFS (broken line) curves after carbon-ion radiotherapy were determined using the Kaplan–Meier method. Abbreviations: OS, overall survival; PFS, progression-free survival

The median follow-up period was 27.9 months (range, 1.5–180.4 months). The 2- and 3-year OS rates were 70.0% (95% confidence interval [CI]: 57.7–79.4%) and 50.2% (95% CI: 37.5–61.7%), respectively. The 2- and 3-year PFS rates were 32.7% (95% CI: 22.0–43.8%) and 20.2% (95% CI: 11.6–30.6%), respectively (Fig. 1). In total, 55 patients were deceased at the last follow-up: 47 patients died of HCC or liver failure and three patients died of aspiration pneumonitis. One patient each succumbed to acute cholecystitis, renal failure, pancreatic carcinoma, aortic dissection, and brain stroke. Overall, 14 patients survived for more than 5 years.

**Fig. 2:**
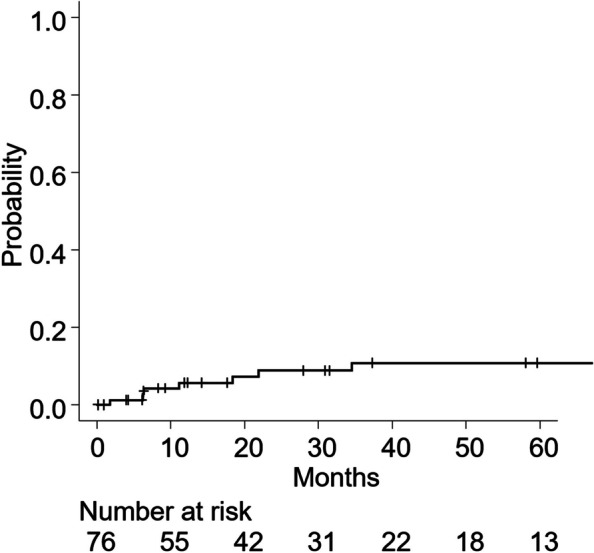
Local recurrence rate curve after carbon-ion radiotherapy. Legend: Local recurrence rate curve after carbon-ion radiotherapy determined using the cumulative incidence function with death as a competing event

The 2- and 3-year LR rates were 8.9% (95% CI: 1.7–23.5%) and 10.7% (95% CI: 2.3–26.6%), respectively (Fig. 2). In total, seven patients developed LR. Furthermore, recurrence occurred in 49 patients (64%), with initial recurrence patterns identified as local in four patients, local + regional in three patients, regional in 37 patients, regional + distant metastasis in two patients, and distant metastasis in three patients.

The results of the univariate and multivariate analyses of prognostic factors for OS are shown in Tables 3 and 4. Naïve tumors and single lesions were significant prognostic factors in univariate analysis, and ALBI grade 1 and single lesions were independent prognostic factors in multivariate analysis. The patient and tumor characteristics of the treatment periods and fraction protocols are shown in Supplementary Tables 3 and 4.

**Table 3:** Univariate analysis of influencing factors of OS

Factor	n	*p*-value	2-year OS rate (%)
Age (years)		0.596	
< 71	37		69.5
≥ 71	39		70.3
Sex		0.056	
Male	58		65.1
Female	18		83.3
Performance status		0.530	
0	55		74.0
1 or 2	21		60.7
Child–Pugh grade		0.107	
A	68		73.3
B	8		42.9
ALBI grade:		0.065	
1	47		79.3
2a or 2b	29		54.4
AFP (ng/mL)		0.885	
< 400.0	51		71.8
≥ 400.0	25		66.8
DCP (mAU/mL)		0.059	
= < 100.0	35		80.9
≥ 100.0	41		61.2
Tumor status		0.016	
Naïve	40		81.4
Reginal or local recurrence	36		56.6
Number of lesions		0.018	
1	64		74.2
≥ 2	12		50.0
Maximum tumor diameter (cm)		0.200	
< 4.6	37		80.5
≥ 4.6	39		59.7
Vp4 or Vv3		0.297	
Yes	17		62.3
No	59		71.8
Protocol dose		0.030	
45.0–48.0 Gy/2 fractions	32		77.5
52.8–60.0 Gy/4 fractions	44		65.1
Start date of C-ion RT		0.133	
First half (1995–2007)	35		68.6
Second half (2008–2020)	41		72.7

*Abbreviations*: *OS* Overall survival, *ALBI* *grade* Albumin-bilirubin grade, *AFP* Alpha-fetoprotein, *DCP* Des-gamma carboxyprothrombin, *C-ion RT* Carbon-ion radiotherapy

**Table 4:** Multivariate analysis of influencing factors of OS

**Factor**	**n**	***p*-value**	**Hazard ratio** **(95% CI)**
Age (years)		0.103	
< 71	37		1.713
≥ 71	39		(0.896–3.274)
Sex		0.302	
Male	58		0.646
Female	18		(0.282–1.481)
Child–Pugh grade		0.451	
A	68		0.658
B	8		(0.221–1.956)
ALBI grade:		0.028	
1	47		0.457
2a or 2b	29		(0.227–0.918)
Tumor status		0.104	
Naïve	40		0.576
Reginal or local recurrence	36		(0.296–1.120)
Number of lesions		0.027	
1	64		0.418
≥ 2	12		(0.193–0.904)
Maximum tumor diameter (cm)		0.202	
< 4.6	37		1.472
≥ 4.6	39		(0.812–2.668)

*Abbreviations*: *OS* Overall survival, *CI* Confidence interval, *ALBI* *grade* Albumin-bilirubin grade

### Toxicity

The acute and late adverse events (grade ≥ 2) of C-ion RT are summarized in Table 5. Only 4 patients (5%) experienced grade 3 late adverse events. No grade 4 or 5 acute or late adverse events were observed.

**Table 5:** Acute and late grade ≥ 2 adverse events

Adverse event	Grade 2	Grade 3	Grade 4	Grade 5
Acute				
Dermatitis	6	2	0	0
Hepatobiliary disorder	4	1	0	0
Pneumonitis	1	0	0	0
Late				
Dermatitis	1	2	0	0
Hepatobiliary disorder	3	2	0	0

## Discussion

In this retrospective cohort study, the 2-year LR and OS rates were 8.9% and 70.0%, respectively, without grade 4 or 5 adverse events, in patients with HCC with MVI treated with C-ion RT. In univariate analysis, naïve tumor, single lesion, and two-fraction protocol were significant factors, and in multivariate analysis, ALBI grade 1 and single lesion were identified as independent significant factors for OS. Although there was no difference in the LC depending on the fraction protocols, the two-fraction protocol was a significant factor. We acknowledge that selection bias may have been influenced by the clinical trials of the two-fraction protocols.

To the best of our knowledge, only one small-scale study has reported C-ion RT for HCC with MVI, and our outcomes are similar to those of this study; the 2-year LR and OS rates were 22% and 64%, respectively, without grade 4 or 5 adverse events [32]. A multicenter prospective registry study on proton beam radiotherapy reported a 3-year OS rate of 21.7% for patients with HCC with portal vein tumor thrombus [33]. Regarding LR in unresectable HCC with MVI, studies of patients treated with conventional photon radiotherapy alone or combination therapy with transcatheter arterial chemoembolization (TACE) have demonstrated that the overall response rate (defined as complete remission + partial remission) is only 40–50%, and unfavorable LR remains a challenge for photon radiotherapy [11–13]. SBRT was performed to reduce the LR rate following conventional radiotherapy techniques. Matsuo et al. reported an overall response rate of 67% and a 1-year LR rate of 20.4% for SBRT for MVI, and both rates were significantly superior to those of conventional photon radiotherapy [34].

A meta-analysis also reported a favorable local response rate to SBRT [35]. SBRT is expected to achieve a lower LR rate than conventional techniques. However, owing to the physical characteristics of photons, SBRT has limited tissue-sparing benefits, particularly for the surrounding normal tissues. SBRT also increases the irradiation dose to the normal liver tissue and the risk of radiation-induced liver disease, especially in large lesions [36]. Thus, SBRT is recommended only for small tumors (generally < 3–5 cm), because of the normal liver constraints. As the median tumor diameter in this study was 4.6 cm, cases with MVI were often large lesions, and it was challenging to adapt SBRT to all cases. This limitation does not apply to particle therapy because of its physical characteristics, including the Bragg peak. Various studies have consistently reported similar or equivalent dose to peripheral lesions with particle therapy, resulting in reduced LR [32, 37–39].

However, reports on C-ion RT for HCC without MVI have shown only a few cases of late grade 3 or severe adverse events [20]. Late grade 3 adverse events were observed in 5% of the patients in our study. The details of these events are as follows: for liver dysfunction after C-ion RT, grade 3 increases in liver-derived enzymes were observed in two patients, but there was no serious hepatic damage deteriorating the Child–Pugh grade. There were no cases of suspected radiation-induced liver disease in contrast to the 5–10% risk associated with photons [26]. Grade 3 dermatitis as a late adverse event is observed in patients treated using passive scattering methods. In contrast, no cases of grade 3 or higher dermatitis as a late adverse event were observed in patients treated with energy scanning. In cases of MVI treated with photon radiotherapy with or without TACE adverse events of grade ≥ 3 accounted for at least 10%, encompassing all adverse events [12, 13]. The incidence of C-ion RT-related late grade 3 adverse events in the current study may be slightly higher than that previously reported [32]. However, this rate is clearly lower than that of photon radiotherapy; thus, we considered it to be acceptable.

For over a decade, sorafenib has been recommended by the AASLD/BCLC staging system and treatment guidelines as the primary treatment modality for HCC with MVI; however, the median survival benefit is only 10 months [2]. This study found no difference in OS before and after 2008, when sorafenib was introduced, partly because the therapeutic effects of sorafenib were limited. In recent years, atezolizumab + bevacizumab combination immunotherapy has prolonged survival to 20 months; however, this is still far from satisfactory compared to the outcomes of early-to intermediate-stage HCC [8]. In our study, the 2-year OS rate was 70%, consistent with favorable results reported by other studies on particle therapy as a radical treatment, with 2-year OS rates ranging from 48 to 88% [32, 37, 38]. Considering that these reports preceded combination immunotherapy, the reduced LR of particle therapy may have contributed to the prolonged OS. This finding is consistent with the successful results of surgical resection in resectable cases of HCC with MVI [9, 10]. However, unlike surgical resection, which requires extensive anatomical resection for advanced disease, particle therapy affects only the tumor and a small surrounding volume; thus, it is more feasible in a wider patient population. Unsurprisingly, the liver function-related adverse events following particle therapy have been minimal [32, 37–39].

However, although the C-ion RT resulted in favorable LR rates, it was still far from satisfactory. The 2-year PFS was only > 33%, which was not surprising considering the systemic nature of HCC with MVI. Thus, despite C-ion RT achieving better results as a local therapy, it should be considered for further improvement. Although radiotherapy has long been known for its immunogenicity, the benefits of combining it with immune checkpoint inhibitors (ICIs) have yet to be proven clinically [40]. Given that C-ion RT is expected to have stronger local immunogenic and immunosuppressive characteristics than photons and protons, combination therapy with ICIs would be interesting [41, 42].

This study has some limitations. First, it had a single-center retrospective design and was thus subject to numerous biases. Although investigator-derived bias was minimized to the fullest extent possible, it was still prone to other biases such as selection. Second, the number of enrolled patients was small, limiting our ability to thoroughly investigate the risk factors affecting the outcome. Although to the best of our knowledge, this is the largest study to date, a prospective study aimed at advanced HCC is required. Third, the patient inclusion spanned more than 20 years, since 1995, and the treatment strategies for HCC and viral hepatitis have changed drastically during this period. Consequently, the outcomes reported in this study may not reflect the current clinical outcomes. Nevertheless, considering the results of this study, any potential bias would likely only push the outcomes downward and, does not affect the value of C-ion RT for these patients. A randomized study with larger patient cohort is warranted to assess the true clinical impact of C-ion RT.

In conclusion, C-ion RT for HCC with MVI resulted in more favorable LR rates and longer survival than those reported in previous studies.

### Supplementary Information

**Supplementary Material 1.**

## Data Availability

The datasets used and/or analyzed during the current study are available from the corresponding author on reasonable request.

## Abbreviations

AASLD/BCLC: American Association for the Study of the Liver Disease/Barcelona Clinic for Liver Cancer
ALBI grade: Albumin-bilirubin grade
C-ion RT: Carbon-ion radiotherapy
CT: Computed tomography
GTV: Gross tumor volume
HCC: Hepatocellular carcinoma
ICIs: Immune checkpoint inhibitors
LR: Local recurrence
MRI: Magnetic resonance imaging
MVI: Macroscopic vascular invasion
OS: Overall survival
PFS: Progression-free survival
PTV: Planning target volume
RBE: Relative biological effectiveness
SBRT: Stereotactic body radiotherapy
TACE: Transcatheter arterial chemoembolization
