# Supplementary material for: Carbon-ion radiotherapy for hepatocellular carcinoma with major vascular invasion: a retrospective cohort study
Source: BMC Cancer. 2024 Mar 26;24:383. doi: 10.1186/s12885-024-12154-4 (PMC10964684; doi:10.1186/s12885-024-12154-4)
Supplement: Supplementary file 1 — Supplementary Material 1. [file 12885_2024_12154_MOESM1_ESM.docx]

**Supplementary table 1.** Tumor status of recurrent lesions and previous treatment of local recurrent lesions (n=36)

| **Characteristic** | **Previous treatment** | **n** |
| --- | --- | --- |
| Reginal recurrence |  | 7 |
| Local recurrence | MWA  RFA  TAE  TACE  TAI+PEI  TAE+PEI  TAE+HAIC  TACE+RFA  TAE+RFA | 2  3  6  8  1  2  2  2  3 |

Abbreviations: MWA, microwave ablation; RFA, radiofrequency ablation; TAE, transcatheter arterial embolization; TACE, transcatheter arterial chemoembolization; TAI, transhepatic arterial infusion; PEI, percutaneous ethanol injection; HAIC, hepatic arterial infusion chemotherapy.

**Supplementary table 2.** Treatment modalities for multiple lesions (n=12)

| **Number of lesions** | **Treatment method** | **N** |
| --- | --- | --- |
| 2 | C-ion RT: 2 lesions  C-ion RT: 1 lesion, RFA: 1 lesion | 4  1 |
| 3 | C-ion RT: 2 lesions, RFA: 1 lesion  C-ion RT: 2 lesions, TAE: 1 lesion  C-ion RT: 1 lesion, TAE: 2 lesions | 1  1  2 |
| 4 | C-ion RT: 1 lesion, RFA: 3 lesions  C-ion RT: 1 lesion, TACE: 3 lesions | 1  2 |

Abbreviations: C-ion RT, carbon-ion radiotherapy; RFA, radiofrequency ablation; TAE, transcatheter arterial embolization; TACE, transcatheter arterial chemoembolization

**Supplementary table 3.** Patient and tumor characteristics between first half and second half

|  | **First half**  **(1995–2007)** | **Second half**  **(2008–2020)** | **p-value** |
| --- | --- | --- | --- |
| Age (years)  median (range) | 68 (50–84) | 72 (45–86) | 0.174 |
| Sex  Male  Female | 8  27 | 10  31 | 1.000 |
| Performance status  0  1 or 2 | 19  16 | 36  5 | 0.002 |
| Child-Pugh grade  A  B | 31  4 | 37  4 | 1.000 |
| ALBI grade:  1  2a or 2b | 21  14 | 26  15 | 0.815 |
| AFP (ng/mL)  median (range) | 50.3 (1.1–140000.0) | 152.1 (1.7–34260.0) | 0.328 |
| DCP (mAU/mL)  median (range) | 242.0 (12.0–47200.0) | 109.0 (16.0–60725.0) | 0.140 |
| Tumor status  Naïve  Reginal or local recurrence | 19  16 | 21  20 | 0.821 |
| Number of lesions  1  ≥2 | 28  7 | 36  5 | 0.529 |
| Maximum tumor diameter (cm)  median (range) | 4.8 (2.1–12.0) | 4.3 (0.5–13.0) | 0.387 |
| Vp4 or Vv3  Yes  No | 7  28 | 10  31 | 0.784 |

Abbreviations: ALBI grade, albumin-bilirubin grade; AFP, alpha-fetoprotein; DCP, des-gamma carboxyprothrombin

**Supplementary table 4.** Patient and tumor characteristics between 2 and 4 fractions protocol

|  | **Two fractions**  **protocol** | **Four fractions**  **protocol** | **p-value** |
| --- | --- | --- | --- |
| Age (years)  median (range) | 74 (45–86) | 66.5 (49–84) | 0.002 |
| Sex  Male  Female | 22  10 | 36  8 | 0.274 |
| Performance status  0  1 or 2 | 27  5 | 28  16 | 0.068 |
| Child-Pugh grade  A  B | 30  2 | 38  6 | 0.455 |
| ALBI grade:  1  2a or 2b | 20  12 | 27  17 | 1.000 |
| AFP (ng/mL)  median (range) | 123.7 (1.7–34260.0) | 64.5 (1.1–140000.0) | 0.453 |
| DCP (mAU/mL)  median (range) | 101.5 (16.0–60725.0) | 263.5 (12.0–47200.0) | 0.270 |
| Tumor status  Naïve  Reginal or local recurrence | 17  15 | 23  21 | 1.000 |
| Number of lesions  1  ≥2 | 27  5 | 37  7 | 1.000 |
| Maximum tumor diameter (cm)  median (range) | 4.25 (0.5–9.6) | 5.0 (2.1–13.0) | 0.021 |
| Vp4 or Vv3  Yes  No | 4  28 | 13  31 | 0.099 |

Abbreviations: ALBI grade, albumin-bilirubin grade; AFP, alpha-fetoprotein; DCP, des-gamma carboxyprothrombin
